# Efficacy and safety of ciprofol-remifentanil versus propofol-remifentanil during fiberoptic bronchoscopy: A prospective, randomized, double-blind, non-inferiority trial

**DOI:** 10.3389/fphar.2022.1091579

**Published:** 2022-12-21

**Authors:** Bin Wu, Wenchao Zhu, Qinghe Wang, Chunguang Ren, Lizhen Wang, Guannan Xie

**Affiliations:** ^1^ Department of Anaesthesiology, Liaocheng People’s Hospital, Liaocheng, China; ^2^ Department of Tuberculosis, Liaocheng Infectious Disease Hospital, Liaocheng, China

**Keywords:** ciprofol, propofol, flexible bronchoscopy, remifentanil, fentanyl

## Abstract

**Objective:** Ciprofol is a novel 2,6-disubstituted phenol derivative that has improved pharmacokinetic and pharmacodynamic properties compared with propofol. This study was conducted to compare the efficacy and safety of ciprofol-remifentanil *versus* propofol-remifentanil for patients undergoing fiberoptic bronchoscopy.

**Methods:** Overall, 92 patients undergoing fiberoptic bronchoscopy were included in this prospective, randomized, double-blind, non-inferiority trial and were equally divided into two groups (n = 46 each). Fentanyl (50 μg) was given 2 min before the intravenous infusion of 0.3 mg/kg of ciprofol or 1.2 mg/kg of propofol over a time period of 30 s. During anesthesia maintenance, 0.05–0.2 μg/kg/min of remifentanil combined with one-third to one-fourth of the initial dose of ciprofol or propofol was repeated at 2-min intervals, as required, to maintain a Modified Observer’s Assessment of Alertness and Sedation (MOAA/S) scale score <3. The primary outcome was the successful rate of fiberoptic bronchoscopy. Secondary outcomes included demographic characteristics, time metrics, hemodynamics, coughing severity, intubating conditions, lowest oxygen saturation, utilization of study drug doses, number of remedies (lidocaine and vasoactive drugs) used, satisfaction scores of both patients and the endoscopist, occurrence of intraoperative awareness, patients’ willing to repeat fiberoptic bronchoscopy, and occurrence and severity of adverse events.

**Results:** The successful completion rate of fiberoptic bronchoscopy was 91.30% (42 of 46; 95% confidence interval [CI]: 82.80%–99.80%) in the ciprofol-remifentanil group and 89.13% (41 of 46; 95% CI: 79.80%–98.50%) in the propofol-remifentanil group. Though the clinically acceptable intubating condition was improved in the ciprofol-remifentanil group, this difference has no clinical statistical difference (*p* > 0.05). No significant differences were noted between the two groups with respect to time metrics, consumption of fentanyl and remifentanil, or number of remedies (lidocaine and vasoactive drugs). Patients’ willingness to repeat fiberoptic bronchoscopy and the satisfaction of both patients and endoscopist were significantly higher in the ciprofol-remifentanil than in the propofol-remifentanil group (*p* < 0.05). Compared with patients in the propofol-remifentanil group, patients in the ciprofol-remifentanil group had more stable hemodynamics. The lowest oxygen saturation was significantly higher in the ciprofol-remifentanil than in the propofol-remifentanil group (*p* < 0.05). The numbers of patients who experienced pain on injection in the ciprofol-remifentanil group was significantly lower than the number in the propofol-remifentanil group (*p* < 0.01). Severity of coughing, clinically acceptable severity of coughing, incidence of intraoperative awareness, and other adverse events were all similar between the two groups (*p* > 0.05). Only four patients experienced grade 2 adverse events (severe hypotension in one patient in the ciprofol-remifentanil group and three patients in the propofol-remifentanil group; *p* > 0.05); they were treated with noradrenaline.

**Conclusion:** Ciprofol-remifentanil was non-inferior to propofol-remifentanil with regard to successful sedation for flexible bronchoscopy, when used with pre-intravenous administration of 50 μg of fentanyl. At the same time, patients’ willingness to repeat flexible bronchoscopy and the satisfactions were all significantly improved.

## 1 Introduction

Fiberoptic bronchoscopy (FB) has gradually become an important diagnostic and therapeutic procedure for the treatment of respiratory diseases. However, this procedure is invasive and painful (Kamel et al., 2020; Wang W. H et al., 2022). As the requirements of patients increase and the diagnostic and treatment procedures using respiratory endoscopy become more complex, sedation and analgesia must be controlled at an optimum level to make this procedure easy to perform (Caron et al., 2021). However, the ideal level of sedation may vary depending on the patient and procedural variables (Cornelissen et al., 2019). As a result, there is still no standard anesthesia protocol for patients undergoing FB.

Total intravenous anesthesia is the most commonly used anesthesia option during FB. Midazolam, ketamine, and dexmedetomidine alone or combined with opioids could be safely used during FB. However, each drug has limitations (Dal et al., 2014; Bi et al., 2019; Lee et al., 2019). Propofol, one of the most widely used intravenous anesthetics throughout the world, has become used more frequently for painless endoscopic therapy (Ashikari et al., 2021). It possesses favorable pharmacokinetic properties with a high clearance rate, rapid onset and recovery, and little residual effects. However, common disadvantages include pain on injection, a narrow therapeutic window, hemodynamic fluctuation, dose-dependent respiratory depression, myoclonus, and lack of antagonists; in addition, it is contraindicated in patients with lipid metabolism disorders, allergic reactions from egg and soybean components. In addition, it can also induce propofol infusion syndrome (Sneyd et al., 2022).

Ciprofol (2-[(1R)-[1-cyclopropylethyl]]-6-isopropylphenol) binds to the α1β2γ2 subtype of the gamma-aminobutyric acid (GABA)-a receptor and has improved pharmacological and physicochemical properties compared with propofol (Qin et al., 2017). It is a promising anesthetic candidate; it has a rapid onset of action and, compared with propofol, has a higher therapeutic index, less respiratory depression, reduced inhibition of cardiac function, and less associated pain on injection, according to the results of several phase I–III trials in China and Australia (Hu et al., 2021; Li et al., 2022; Liu et al., 2022). However, limited data are available regarding its use during FB (Luo et al., 2022). We designed this prospective, randomized, double-blind, non-inferiority trial to explore the efficacy and safety of ciprofol-remifentanil *versus* propofol-remifentanil during FB.

## 2 Materials and methods

### 2.1 Patients

This prospective, randomized, double-blind, non-inferiority trial was carried out at Liaocheng People’s Hospital. The institutional review boards of Liaocheng People’s Hospital approved this study protocol (No. 2022180), and written informed consent was obtained from all patients or their legally authorized representatives before the start of the procedure. This trial was also registered in the Chinese Clinical Trial Registry (ChiCTR2200062763).

Overall, 92 patients who underwent FB from August 2022 to November 2022 were recruited in our hospital. Inclusion criteria were FB with sedation and without endotracheal intubation or mechanical ventilation, patient age of 45–65 years, American Society of Anesthesiologists (ASA) grade I–II, and oxygen saturation (SpO_2_) > 93% under air conditions. Exclusion criteria were a QTc interval ≥450 ms; receipt of drugs within the 2 weeks before the procedure that could affect the QT interval or induce/inhibit cytochrome P450 (particularly CYP2B6) enzymes; history of alcohol/drug abuse; previous anesthesia incidents or nasopharyngeal surgery; known allergies to eggs, soy products, or the test drugs (propofol, ciprofol, remifentanil, or midazolam); body mass index >30 kg/m^2^; difficult airway; pregnancy or lactation; the presence of central nervous system diseases, severe hypertension, diabetes, or liver and kidney dysfunction; refusal to sign the consent forms; participation in other clinical trials in the 3 months prior to the FB; a procedure time >30 min; and the inability to communicate or cooperate.

### 2.2 Randomization and blinding

The randomization schedule was generated by computer with an anesthesiologists. Patients were randomly assigned to two equally groups according to the screening number. Another anesthesiologist signed the informed consent, prepared the drug, and assessed the outcomes. All FB procedures were performed by the same endoscopist and anesthesiologist. Patients, the endoscopist, and the anesthesiologist were all blinded to this trial. The group allocations were unblinded after the trial.

### 2.3 Anesthesia

Patients fasted for at least 8 h and avoided clear fluids for at least 2 h before FB (Green et al., 2020). Electrocardiogram, heart rate, non-invasive blood pressure, peripheral capillary oxygen saturation (i.e., SpO_2_), end-tidal carbon dioxide, and respiratory rate were monitored for all patients. Intravenous access was established by placing a 20-gauge cannula in the right forearm vein; then, patients received 5 ml/kg of 0.9% sodium chloride solution before sedation, according to the methods of a previous study (Yao et al., 2019). All patients were provided with mask oxygen at a rate of 5 L/min until fully awake after airway nebulization with 10 ml of 1% lidocaine. The Modified Observer’s Assessment of Alertness and Sedation (MOAA/S) scale was used to evaluate the level of sedation every 30 s until induction was successful and then every 3 min until the end of the procedure. Hemodynamics were also recorded every 3 min during the procedure.

Patients received 50 μg of fentanyl 2 min before the intravenous infusion of 0.3 mg/kg ciprofol or 1.2 mg/kg propofol over a time period of 30 s. FB was started when the MOAA/S score was <3. If the MOAA/S score remained >3 after 1 min, one-third of the initial dose was injected over 30 s. If this procedure was still not effective, rescue midazolam was administered at the discretion of anesthesiologist, and the induction was deemed to be failed. During anesthesia maintenance, 0.05–0.2 μg/kg/min of remifentanil was administered. One-third to one-fourth of the initial dose of ciprofol or propofol was given to maintain a MOAA/S score <3. Sedation was regarded as unsuccessful if more than five top-up doses were given within 15 min. Midazolam was the only permitted alternative sedative in this trial. In addition, 25 µg of fentanyl was permitted once (maximum, 150 µg) until adequate analgesia could be achieved. The use of topical anesthetics during FB is referred to in our previous research (Chen et al., 2022a). In brief, 10 ml of 1% lidocaine was sprayed over the vocal cords, trachea, and right and left main bronchi to inhibit the patient’s cough reflex. Rescue lidocaine was given at the discretion of the endoscopist, and the total dose of lidocaine never exceeded 5 mg/kg. All patients were transferred to the post-anesthesia care unit after the procedure and were then returned to the ward until a modified Aldrete score of ≥9 was achieved.

### 2.4 Outcomes

The primary outcome was the success rate of FB. The second outcomes were demographic characteristics, time metrics (induction time, procedure time, awake time, and recovery time), hemodynamics (at T1: arrival at the examination room; T2: immediately before start of ciprofol or propofol; T3: immediately after entering the glottis; T4: 3 min after start of entering the glottis; T5: 6 min after start of entering the glottis; T6: end of FB; T7: 3 min after FB; T8: 6 min after FB; and T9: before leaving the post-anesthesia care unit), intubating conditions, use of study drug doses, number of remedies (lidocaine and vasoactive drugs), satisfaction scores of both patients and the endoscopist (on 5-point scales: 1 = dissatisfied, 5 = satisfied), and patients’ willingness to repeat FB.

Safety was assessed by the rate of occurrence of adverse events (AEs). For hypoxemia (oxygen saturation <90% lasting for >30 s), the following measures were taken: increase in oxygen flow, verbal and tactile stimulation, chin lift, jaw thrust, face mask, manual ventilation, and tracheal intubation).Bradycardia (heart rate <50 beats/min lasting for >30 s) was treated with 0.2–0.4 mg of atropine. Hypotension, defined as systolic blood pressure (SBP) < 90 mmHg or a decrease by 30% from the baseline value, lasting for >30 s was treated with 8 μg of noradrenaline. Pain on injection and electrocardiogram assessments were also noted. The severity of AEs was graded according to the National Cancer Institute Common Terminology Criteria for the Classification of Adverse Events (CTCAE) version 5.0. Grade 1 (mild) reflected asymptomatic status or mild symptoms and required clinical or diagnostic observations only (intervention not indicated). Grade 2 (moderate) reflected minimal symptoms with local or non-invasive intervention indicated; these symptoms may have limited age-appropriate instrumental activities of daily living. Grade 3 (severe or medically significant but not immediately life threatening) reflected disabling symptoms that limited self-care and activities of daily living or that required hospitalization or prolongation of hospitalization. Grade 4 reflected events with life-threatening consequences for which urgent intervention was indicated. Grade 5 reflected death related to the AE (Teng et al., 2021a). Severity of coughing was graded as follows: grade 0 (severe), ≥5 coughs; grade 1 (moderate), 3–4 coughs; grade 2 (minimal), 1–2 coughs; and grade 3, no coughing. The lowest oxygen saturation and the occurrence of intraoperative awareness (using a modified Brice questionnaire with a 5-point Likert scale) were also recorded.

### 2.5 Statistical analysis

We assumed that the success rates of sedation from both ciprofol-remifentanil and propofol-remifentanil would be 88%, according to the result of our preliminary study. With a non-inferiority margin of 20% on the relative scale between groups for the primary endpoint, a power of 80%, and a one-sided alpha of 2.5%, the total sample size needed was 82. Assuming a dropout rate of 10%, 46 patients were recruited in each group as a minimum population size.

Statistical analysis was performed using SPSS software 24.0 (SPSS Inc., Chicago, IL, United States). Shapiro–Wilk and Levene tests were used to check the distribution and homogeneity of data. Continuous outcomes were presented as means ± standard deviations or medians and interquartile ranges, and they were analyzed with the Student’s t-test or Kolmogorov–Smirnov *Z* test. Repeated-measures analysis of variance was used to assess hemodynamic measurements. Qualitative data are presented as numbers and frequencies and are analyzed with χ2 or Fisher’s exact tests. *p* < 0.05 was considered statistically significant.

## 3 Results

### 3.1 Patient demographic characteristics

**FIGURE 1:**
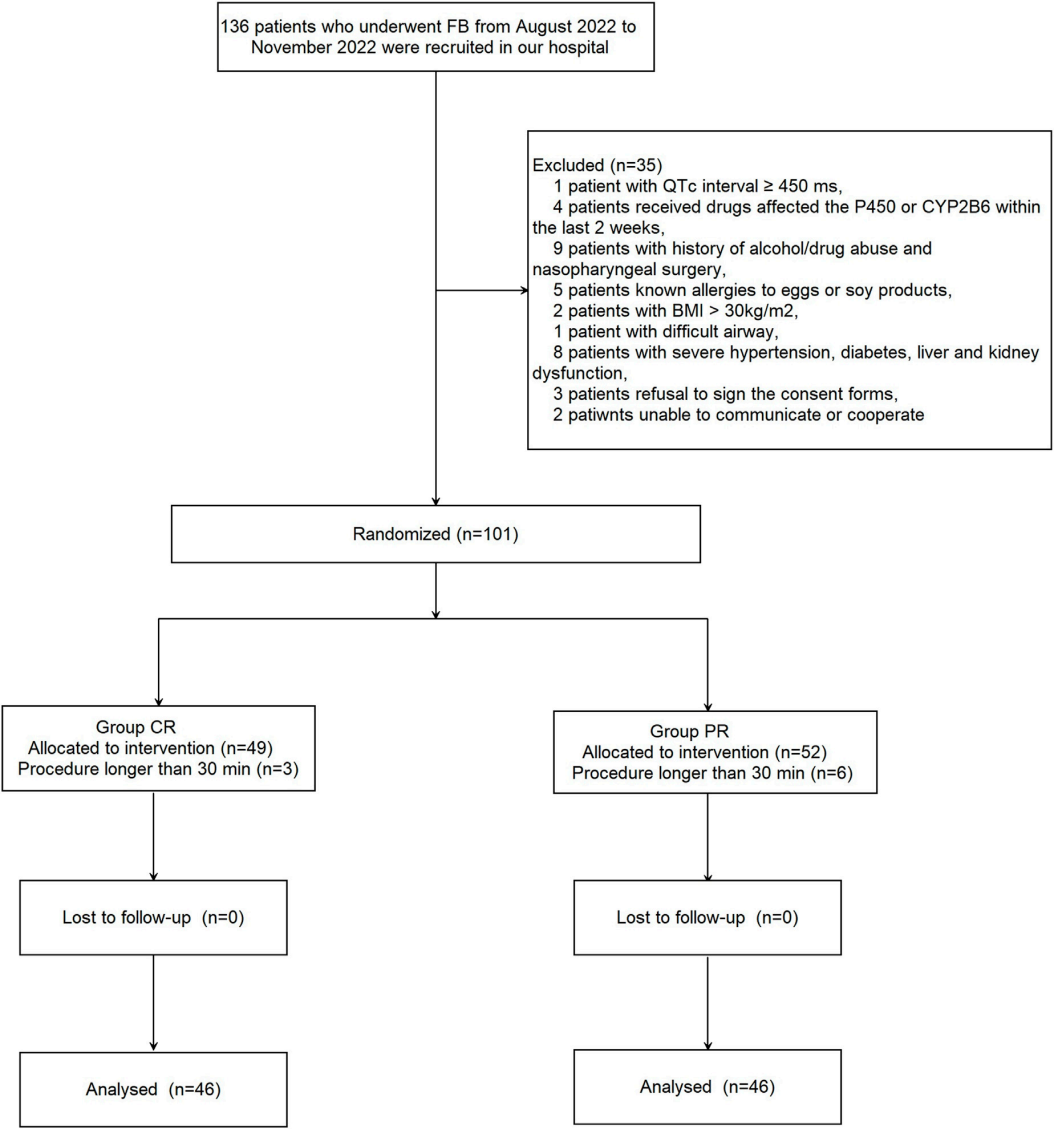
Patient flowchart with CONSORT guidelines.

Overall, 136 patients who underwent FB from August 2022 to November 2022 were recruited in our hospital. Thirty-five patients were excluded from this study for the following reasons: QTc interval ≥450 ms (n = 1); receipt of drugs that may have affected the CYP450 enzymes, especially CYP2B6, within the last 2 weeks (n = 4); history of alcohol/drug abuse and nasopharyngeal surgery (n = 9); known allergies to eggs or soy products (n = 5); body mass index >30 kg/m^2^ (n = 2); difficult airway (n = 1); severe hypertension, diabetes, or liver and kidney dysfunction (n = 8); refusal to sign the consent forms (n = 3); and inability to communicate or cooperate (n = 2). The procedure time of nine patients was >30 min (n = 3 patients in the ciprofol-remifentanil group and n = 6 patients in the propofol-remifentanil group). Ultimately, 92 patients were equally randomly assigned into two groups (Figure 1). No significant differences between the two groups were noted with respect to patients’ demographic characteristics (*p* > 0.05; Table 1).

**TABLE 1:** Demographic characteristics between the two groups.

Variable	Group CR (n = 46)	Group PR (n = 46)	*p*-value
Age (years)	58.02 ± 5.47	57.48 ± 5.28	0.629
Sex (male/female)	26/20	24/22	0.834
History of smoking, n (%)	24 (52.17%)	20 (43.48%)	0.862
FEV1/FVC(%)	87.30 ± 3.47	86.915 ± 3.39	0.587
Height (cm)	167.15 ± 5.75	165.89 ± 5.53	0.287
Body weight (kg)	67.87 ± 6.91	67.39 ± 6.71	0.737
BMI (kg/m^2^)	24.26 ± 1.75	24.49 ± 2.09	0.573
ASA I/Ⅱ (n)	10/36	8/38	0.793
Comorbidity, n (%)			1.000
Hypertension	15 (32.61%)	16 (34.78%)	
Diabetes	8 (17.39%)	7 (15.22%)	
Coronary heart disease	7 (15.22%)	6 (13.04%)	
Indication, n (%)			0.975
Lung cancer	22 (47.83%)	20 (43.48%)	
Pneumonia	15 (32.61%)	16 (34.78%)	
Pulmonary tuberculosis	5 (10.87%)	5 (10.87%)	
Others	4 (8.70%)	5 (10.87%)	
Procedure, n (%)			0.587
Endobronchial inspection	5 (10.87%)	6 (13.04%)	
Bronchoscopic biopsy	26 (56.52%)	21 (45.65%)	
Bronchoalveolar lavage	15 (32.61%)	19 (41.30%)	

Variables presented as mean ± SD or number of patients n (%). FEV1, forced expiratory volume in the first second; FVC, forced vital capacity; BMI, body mass index; ASA, american society of anesthesiology.

### 3.2 Efficacy

#### 3.2.1 Primary outcome

The successful completion rate of FB was 91.30% (42 of 46; 95% confidence interval [CI]: 82.80%–99.80%. 3 patients needed midazolam during the induction of anesthesia, 1 patient needed midazolam during the maintenance of anesthesia) in the ciprofol-remifentanil group and was 89.13% (41of 46; 95% CI: 79.80%–98.50%. 3 patients needed midazolam during the induction of anesthesia, 2 patients needed midazolam during the maintenance of anesthesia) in the propofol-remifentanil group. The difference in the successful completion rate of FB between the two groups was 2.17% (95% CI: 1.30%–3.00%). As a result, because the higher limit of the 95% CI for the difference in the successful completion rate of FB was not greater than the non-inferiority limit of 20%, ciprofol-remifentanil was considered non-inferior to propofol-remifentanil in patients undergoing FB (Table 2).

**TABLE 2:** The primary outcome in this study.

Variable	Group CR (n = 46)	Group PR (n = 46)	*p*-value
Procedure success, n (%)	42 (91.30%)	41 (89.13%)	1.000
95% CI	(82.80%, 99.80%)	(79.80%, 98.50%)	
Difference in rates	2.17%		
95% CI	(1.30%, 3.00%)		

Variables presented as number of patients n (%). CI, confidence interval.

#### 3.2.2 Secondary outcomes

The induction time (0.64 ± 0.27 vs. 0.62 ± 0.26 min), procedure time (17.98 ± 5.57 vs. 18.09 ± 6.20 min), awake time (4.70 ± 1.43 vs. 4.67 ± 1.94 min), and recovery time (11.43 ± 2.94 vs.11.22 ± 2.79 min) were all comparable between the two groups (*p* > 0.05, Table 3). Though the clinically acceptable intubating conditions (excellent and good) were improved in the ciprofol-remifentanil group compared with the propofol-remifentanil group, this difference was not significantly different (*p* > 0.05, Table 3) (Ghezel-Ahmadi et al., 2015). There were also no significant differences between the two groups with respect to the consumption of fentanyl and remifentanil or the number of remedies (lidocaine and vasoactive drugs; *p* > 0.05, Table 3). The satisfaction of both patients and the endoscopist were significantly higher in the ciprofol-remifentanil group than in the propofol-remifentanil group (*p* < 0.01, Table 3). Patients’ willingness to repeat FB with the same anesthesia scheme was also higher with ciprofol-remifentanil than with propofol-remifentanil (91.30% vs. 73.91%, *p* = 0.028, Table 3).

**TABLE 3:** The secondary outcomes in this study.

Variable	Group CR (n = 46)	Group PR (n = 46)	*p*-value
Time metrics			
Induction time (min)	0.64 ± 0.27	0.62 ± 0.26	0.697
Procedure time (min)	17.98 ± 5.57	18.09 ± 6.20	0.930
Awake time (min)	4.70 ± 1.43	4.67 ± 1.94	0.951
Recovery time (min)	11.43 ± 2.94	11.22 ± 2.79	0.717
Intubating conditions, n (%)			0.195
Excellent	19 (41.30%)	13 (28.26%)	
Good	22 (47.83%)	22 (47.83%)	
Poor	5 (10.87%)	11 (23.91%)	
Consumption of fentanyl (µg)	60.87 ± 20.17	60.33 ± 18.69	0.894
Consumption of remifentanil (µg)	117.00 ± 26.42	120.72 ± 28.58	0.518
Consumption of ciprofol (mg)	48.00 (40.00–55.25)	—	
Consumption of propofol (mg)	—	180.00 (140.00–240.00)	
Number of remedies lidocaine, n (%)	14 (30.43%)	10 (21.74%)	0.477
Number of vasoactive drugs, n (%)	7 (15.22%)	9 (19.57%)	0.784
Satisfaction of patients	5.00 (4.00–5.00)	4.00 (4.00–5.00)*	0.006
Satisfaction of endoscopist	4.00 (4.00–5.00)	4.00 (3.00–5.00)*	0.005
Willing to the repeat bronchoscopy, n (%)	42 (91.30%)	34 (73.91%)*	0.028

Variables presented as mean ± SD, median (interquartile range) or number of patients n (%). *p < 0.05 vs. Group CR.

### 3.3 Safety

**FIGURE 2:**
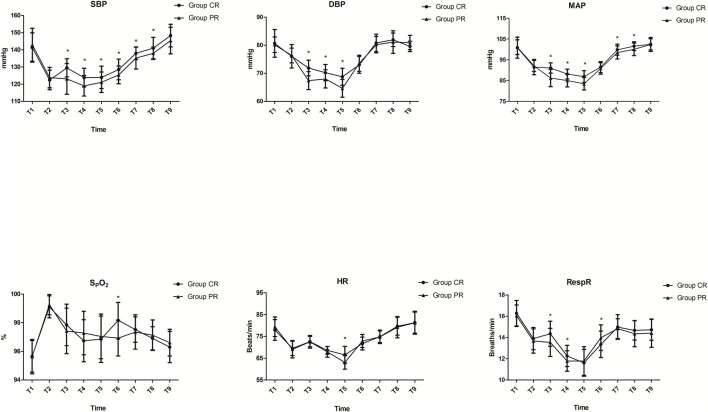
Hemodynamic measurements. Systolic blood pressure (SBP) was significantly higher from T3 to T8, whereas diastolic blood pressure (DBP) values declined significantly less from T3 to T5 in the ciprofol-remifentanil (CR) group (*p* < 0.05). Mean arterial blood pressure (MAP) was also significantly higher in the CR group from T3 to T8, except during T6 (*p* < 0.05). SpO_2_ and heart rate (HR) were significantly decreased at T6 and T5, respectively, in the propofol-remifentanil (PR) group, and the respiratory rate (RespR) was significantly increased at T3, T4, and T6 in the CR group (*p* < 0.05). Time metrics are as follows: T1: arrival at the examination room; T2: immediately before start of ciprofol or propofol; T3: immediately after entering the glottis; T4: 3 min after start of entering the glottis; T5: 6 min after start of entering the glottis; T6: end of fiberoptic bronchoscopy (FB); T7: 3 min after FB; T8: 6 min after FB; T9: before leaving the postanesthesia care unit (PACU).

Compared with the blood pressure measures in the propofol-remifentanil group, systolic blood pressure was significantly higher from T3 to T8 in the ciprofol-remifentanil group, but diastolic blood pressure values declined significantly less from T3 to T5 that group (*p* < 0.05, Figure 2). The mean arterial blood pressure was also significantly higher in the ciprofol-remifentanil group than in the propofol-remifentanil group from T3 to T8, except during T6 (*p* < 0.05, Figure 2). The SpO_2_ and heart rate were significantly decreased at T6 and T5, respectively, in the propofol-remifentanil group. The respiration rate was significantly increased at T3, T4, and T6 in the ciprofol-remifentanil *versus* the propofol-remifentanil group (*p* < 0.05, Figure 2). Similarly, the lowest oxygen saturation was significantly higher in the ciprofol-remifentanil group *versus* in the propofol-remifentanil group (90.09% ± 2.55% vs. 88.83% ± 1.96%, *p* = 0.009, Table 4).

**TABLE 4:** The Safety parameters between two groups.

Variable, n (%)	Group CR (n = 46)	Group PR (n = 46)	*p*-value
Lowest oxygen saturation (%)	90.09 ± 2.55	88.83 ± 1.96*	0.009
Severity of coughing, n (0/1/2/3)	4/15/17/10	5/14/16/11	1.000
Incidence of intraoperative awareness, n (%)	19 (41.30%)	17 (36.96%)	0.831
Pain on injection	3 (6.52%)	17 (36.96%)*	0.001
Hypotension	5 (10.87%)	12 (26.09%)	0.060
Hypertension	4 (8.70%)	4 (8.70%)	1.000
Bradycardia	3 (6.52%)	5 (10.87%)	0.714
Hypoxia	9 (19.57%)	12 (26.09%)	0.456
Respiratory depression	4 (8.70%)	5 (10.87%)	1.000
Arrhythmia	5 (10.87%)	3 (6.52%)	0.714
Epistaxis	2 (4.35%)	1 (2.17%)	1.000
Severity of adverse events			0.617
Grade 1	45 (97.83%)	43 (93.48%)	
Grade 2	1 (2.17%)	3 (6.52%)	
Grade 3	0	0	
Grade 4	0	0	

Variables presented as mean ± SD or number of patients n (%). *p < 0.05 vs. Group CR.

Severity of coughing, clinically acceptable severity of coughing (grade ≥1), and incidence of intraoperative awareness were also similar between the two groups (*p* > 0.05, Table 4). Nine patients (19.57%) in the ciprofol-remifentanil group and 12 patients (26.09%) in the propofol-remifentanil group had hypoxia and needed increased oxygen delivery; four patients (8.70%) in the ciprofol-remifentanil group and five (10.87%) in the propofol-remifentanil group required a jaw thrust maneuver (*p* > 0.05, Table 4). The number of patients who experienced pain on injection in the ciprofol-remifentanil group was significantly decreased compared with that of the propofol-remifentanil group (6.52% vs. 36.96%, *p* < 0.01, Table 4). The incidences of hypotension (10.87% vs. 26.09%), hypertension (8.70% vs. 8.70%), bradycardia (6.52% vs. 10.87%), and arrhythmia (10.87% vs. 6.52%) were similar in the ciprofol and propofol groups. Epistaxis was recorded in three patients (n = 2 patients in the ciprofol-remifentanil group and n = 1 patient in the propofol-remifentanil group, *p* > 0.05, Table 4). Only four patients experienced grade 2 AEs, which were all severe hypotension (n = 1 patient in the ciprofol-remifentanil group and n = 3 patients in the propofol-remifentanil group); all four patients were treated with noradrenaline (*p* > 0.05, Table 4).

## 4 Discussion

In this trial, we found that ciprofol-remifentanil was non-inferior to propofol-remifentanil with regard to successful sedation of FB after the pre-intravenous administration of 50 μg of fentanyl. The number of patients who experienced pain on injection was significantly reduced, and the hemodynamics were more stable in the ciprofol-remifentanil group. In addition, the lowest oxygen saturation, patients’ willingness to repeat FB, and the satisfaction of both patients and the endoscopist were significantly higher in the ciprofol-remifentanil group than in the propofol-remifentanil group.

Since the late 1980s, propofol has been the commonly used intravenous anesthetic drug, because of its fast onset, fast clearance, and rapid patient recovery. However, propofol has unavoidable limitations, such as a narrow therapeutic index, injection pain, circulation and respiratory depression, and infusion syndrome (Hughes et al., 2021). The active ingredient of ciprofol is similar to propofol, but it has single R-configured diastereoisomers (Liao et al., 2022). The innovation of this drug lies in the cyclopropyl group, which not only increases the steric effect but also introduces stereoselective effects over their anesthetic properties (Li et al., 2021; Hu et al., 2022). A previous study has reported that 0.4–0.9 mg/kg of ciprofol was well tolerated, induced dose-dependent sedation and general anesthesia, and had rapid onset and recovery (Teng et al., 2021b). Another study comparing ciprofol (0.5 mg/kg) and propofol (2.0 mg/kg) showed that the duration of colonoscope insertion in two group was similar; the ciprofol group had a slightly shorter duration (Ghezel-Ahmadi et al., 2015). However, another study revealed that a dose of 0.3 mg/kg of ciprofol was similarly efficacious in the elderly when compared with 0.4 mg/kg in non-elderly patients because of the physiological changes that occur in elderly patients (Ding et al., 2022). Although the pharmacokinetic characteristics of 0.4 mg/kg of ciprofol are similar in elderly and non-elderly patients in some studies, this trial used 0.3 mg/kg of ciprofol to account for a variety of patient factors, procedure differences, and anesthesia schemes (especially the combination with remifentanil). Previous phase I–III clinical trials have reported that only 20%–25% of a ciprofol dose was needed to achieve the same anesthetic effect as propofol (Hu et al., 2021; Li et al., 2022; Liu et al., 2022). Thus, this trial used 1.2 mg/kg propofol during anesthesia induction. The results of one phase II clinical trial concluded that the recommended initial maintenance dose of ciprofol is 0.8 mg/kg/h in restricted patients undergoing elective surgery (Qin et al., 2022). However, the protocol of this trial adopted intermittent injections, in consideration of the time of the procedure and the medical expense for the patients. In this trial, ciprofol-remifentanil was non-inferior to propofol-remifentanil with regard to successful sedation of FB after pre-intravenous administration of 50 μg of fentanyl (91.30% vs. 89.13%). The incidence of successful sedation of FB was lower than that of the previous study, which may be due to the different levels of sedation adopted (MOAA/S score ≤1 previously vs. MOAA/S score <3 in this study), different anesthesia management, and lower induction doses of ciprofol/propofol (Luo et al., 2022). Though the clinically acceptable intubating conditions were improved in the ciprofol-remifentanil group, this difference has no clinical statistical difference.

We only recruited patients with ASA I-II and those who had a procedure time <30 min (eg, endobronchial inspection, bronchoscopic biopsy, and bronchoalveolar lavage). However, with the development of respiratory endoscopy technology, more complex endoscopic therapy is required under anesthesia, when there is no contraindication according to guidelines (Wahidi et al., 2011; Strohleit et al., 2021; Long et al., 2022). A previous study reported that ciprofol produces similar levels of sedation compared with propofol in the intensive care unit setting to achieve required sedation times of 6–24 h (Liu et al., 2022). This finding is particularly beneficial for patients who need long-term sedation, because propofol is associated with an increased risk of hypertriglyceridemia as a result of its formulation—a 10% oil-in-water lipid emulsion (Pancholi et al., 2022). More research should explore the optimal maintenance dose of ciprofol, especially for long-term sedation during FB.

Ciprofol may inhibit a wide range of CYP450 isozymes in mammalian species, so 4 patients were excluded because they received drugs that may affect CYP450 enzymes, particularly CYP2B6, within 2 weeks before the FB (Bian et al., 2021). Ciprofol has a minor effect on the cardiovascular and respiratory system because of peripheral vasodilation, reduction in ventricular preload, sympathetic activity, and myocardial contractility. A previous study reported that patients in a ciprofol group, compared with patients in a propofol group, had fewer intubation responses and fewer occurrences of bispectral index (BIS) > 60 within 15 min of intravenous administration, which indicated that ciprofol may provide a better sedation level than propofol during the induction period (Wang X et al., 2022). Consistent with these results, in this trial, the hemodynamics were more stable and the lowest oxygen saturation was higher for patients in the ciprofol-remifentanil group. Inconsistent with the results of the previous study, the time metrics in the ciprofol-remifentanil group in this trial were similar to those in the propofol-remifentanil group. Such a discrepancy could be attributed to different procedure and anesthesia schemes (Chen et al., 2022b). The changes in liver and kidney functions observed patients before and after FB should be documented, as well, because propofol is mainly metabolized in the liver, but ciprofol is mainly metabolized through the kidney (Chen et al., 2022c). However, we did not recorded the statistical difference which partly because of the fewer sample size and the patients with ASA I–II in this trial.

Pain on injection is one of the most common adverse reactions of propofol; it may cause anxiety, discomfort, body movements, and resistance in patients. The overall incidence of injection pain in adults is approximately 50%–80% and can be as high as 90% in children (Habre et al., 2014; Bakhtiari et al., 2021). The judgment of injection pain may be affected in this trial by the use of 50 μg of fentanyl 2 min before the start of sedative drugs, and the analgesic effect of remifentanil during the procedure. As such, the incidence of pain on injection in the propofol-remifentanil group was lower in this trial than in prior reports. Consistent with the results of previous research, pain on injection in the ciprofol-remifentanil group was low compared with pain reported by patients in the propofol-remifentanil group (6.52% vs. 36.96%) (Ding et al., 2022); one reason for this difference is the lower concentration of drug in the aqueous phase of the emulsion. The greater hydrophobicity and lower plasma concentration of ciprofol may also contribute to the lower rates of injection pain *versus* propofol (Nair and Seelam, 2022). Satisfaction of both patients and endoscopist and patients’ willingness to repeat FB were higher in the ciprofol-remifentanil group than in the propofol-remifentanil group, which may also be due in part to the lower incidence of pain on injection. Inconsistent with the results of a previous study, the incidence of AEs, except pain on injection, was similar between the two groups (Li et al., 2022). These differences may be due to small sample sizes in our trial and different anesthesia schemes. However, 4 patients still experienced grade 2 AEs of severe hypotension (1 patient in the ciprofol-remifentanil group and 3 patients in the propofol-remifentanil group); all 4 patients were treated with noradrenaline.

This trial has the following limitations. First, this trial was conducted in patients with ASA I–II. Whether this conclusion can be extrapolated to patients with ASA III or IV requires additional investigation. Second, the level of sedation was adopted according to a subjective scale. Combination of both subjective and objective indicators may provide a more accurate level of sedation for patients undergoing FB. Third, both ciprofol and propofol were injected intermittently, but continuous intravenous infusion may be more beneficial to patients undergoing FB. Finally, this study was a small sample, single-center trial, and these preliminary results must be validated by a prospective, multicenter study with a larger sample size.

In conclusion, ciprofol-remifentanil was non-inferior to propofol-remifentanil with regard to successful sedation for FB after pre-intravenous administration of 50 μg fentanyl. At the same time, patients’ willingness to repeat flexible bronchoscopy and the satisfactions were all significantly improved. Ciprofol combined with remifentanil may be a new anesthesia option for FB.

## Data Availability

The original contributions presented in the study are included in the article/supplementary material, further inquiries can be directed to the corresponding author.
